# Improving Effective Surgical Delivery in Humanitarian Disasters: Lessons from Haiti

**DOI:** 10.1371/journal.pmed.1001025

**Published:** 2011-04-26

**Authors:** Kathryn Chu, Christopher Stokes, Miguel Trelles, Nathan Ford

**Affiliations:** 1 Médecins sans Frontières, Cape Town, South Africa; 2 Médecins sans Frontières, Brussels, Belgium; 3 Médecins sans Frontières, Geneva, Switzerland

## Abstract

Kathryn Chu and colleagues describe the experiences of Médecins sans Frontières after the 2010 Haiti earthquake, and discuss how to improve delivery of surgery in humanitarian disasters.

**Box 1.** Recommendations for the Next Disaster: The Role of the Emergency Surgery Coalition

**Emergency Surgery Coalition (ESC):** Organizations with extensive experience in delivering surgical care in humanitarian emergencies could work together as an ESC to support the rapid deployment of emergency surgical services.

***BEFORE THE DISASTER***

- **Emergency prep kits.** The ESC would maintain emergency supplies worldwide. Ideally, small-scale projects in “hotspots” would be maintained in order to establish local relationships and supply chains.
- **Prior import agreements.** The ESC would work with governments and the World Health Organization to minimize administrative delays to the importation of surgical, anesthetic, and related medical supplies.
- **Training of surgical personnel.** The ESC would facilitate surgical training for humanitarian settings and share lists of qualified personnel among its members.
- **Define a surgical mass disaster plan.** When thousands of victims need surgical attention, scarce surgical resources need to be directed according to agreed priorities.

***DURING THE DISASTER***

- **Inter-agency coordination:** The ESC would coordinate emergency and referral care in a manner that reduces duplication and fragmentation between providers.
- **Supply delivery.** The ESC would work with government and the World Health Organization to ensure the timely delivery of humanitarian supplies according to collective needs assessments.

***AFTER THE DISASTER***

- **Monitoring and evaluation.** ESC members would use a standardized database to collect data on demographics and procedures to analyze the quality and expediency of the surgical care delivered. Lessons learned would be shared among members to prepare for the next disaster.

The humanitarian response to major disasters is often marred by duplication and fragmentation, resulting in insufficient resources and services reaching the victims [1]. This is particularly critical when it comes to surgical care in mass disasters, both because the impact of surgical services on mortality requires a rapid response, and because surgical teams are often the most difficult to recruit.

In response to the 2010 Haiti earthquake, Médecins sans Frontières (MSF) deployed the largest surgical team in the organization's 40-year history: in 10 weeks, over 55,000 patients were treated and over 4,000 surgical interventions performed. The overall combined response was perhaps one of the largest non-conflict humanitarian surgical efforts in human history. However, the delivery of care was fraught with supply delays, a lack of appropriately experienced surgeons and anesthesiologists, and challenges in coordinating with other agencies—governmental, military, and non-governmental—whose priorities and motives did not always agree. We highlight some challenges from this recent experience and propose some ways forward to support an effective surgical humanitarian response to future major disasters.

## Improving Collaboration between Relevant Services

Surgical care was one of the primary health needs in the post-earthquake response. An estimated quarter of a million injured victims suffered internal injuries, crushed limbs, open wounds, and fractures [2]. The most gravely injured victims died instantly or shortly after the quake due to long extraction times and the severity of their wounds. First-aid and triage stations were essential in the first hours. Many patients needed surgical procedures such as wound debridement and amputations—these operations were required within days. In the ensuing weeks, surgical needs increased as more patients sought medical care, often with infected, even gangrenous wounds. Non-trauma surgical needs such as emergency obstetrical care escalated in the weeks to months after the quake as the pre-earthquake fragile health care system completely collapsed.

Over 600 health agencies responded to the Haiti earthquake, but few had the relevant experience, competence, or capacity to provide the infrastructure needed to support emergency surgical services. A lack of coordination of services resulted in too many agencies trying to provide the same care in the same area while other sections of the city had no access to emergency care. Many military and humanitarian groups left after a few weeks, leaving thousands of post-operative patients behind. MSF's post-operative and rehabilitation hospitals were overwhelmed with patients who had been left without follow-up for their amputated limbs, wounds, and fractures. Referral systems were not well established. Communication between agencies was poor and most worked in isolation from one another. Standard databases and common definitions were not shared between agencies and the total number of operations and interventions performed during the months after the earthquake is unknown. Rumors circulated by media about inappropriate amputations [3] were difficult to confirm or refute without reliable data.

Criticism of poor coordination is a common feature of large humanitarian disasters from the Rwandan genocide [4] to the 2004 Asian tsunami [5], and Haiti was no different [1],[6]. The need for improved coordination between humanitarian actors in emergencies is a long-standing concern: efforts to formalize international oversight began with proposals to establish a relief coordination function as part of the United Nations (UN) system in the mid-1960s. Specifically, in 1991, the Office for the Coordination of Humanitarian Affairs (OCHA) was created to improve inter-agency coordination [7]. In 2005, the cluster system was established to provide information to organizations delivering care in different sectors such as health, water and sanitation, and food and logistics. However, relief efforts in high-profile emergencies generate significant media and political attention and financial support, leading to a mass influx of actors—over 2,000 agencies responded to the Haiti earthquake according to some estimates. The presence of such a high volume of actors with varying motives, competencies, and specificities inevitably results in a degree of competition and duplication among actors. Many smaller non-governmental organizations could not function independently and drained UN resources through demands for logistical assistance [8]. Lack of coordination is only part of the problem.

## Proximity

Proximity to the disaster is paramount to responding rapidly and reducing the extremely high mortality that often occurs in the immediate aftermath of a disaster. While disasters are by definition unpredictable, experienced agencies can identify a shortlist of hotspot regions that are prone to conflicts (e.g., central Africa) or natural disasters such as earthquakes and cyclones (e.g., Central America) and establish small-scale medical projects in order to maintain local networks and emergency supplies of materials and medications. This presence in Haiti prior to the earthquake allowed MSF and other actors to respond within hours, just as the International Committee for the Red Cross (ICRC) was among the first to deliver care during the 2005 earthquake in Pakistan. In contrast, few agencies were present in China prior to the 2008 Sichuan earthquake and the humanitarian surgery response there was less effective.

The ability to rapidly import essential materials is another challenge. MSF's ability to respond in the immediate aftermath of the Haiti earthquake was assisted by pre-positioned stock held in Panama that arrived in Haiti the day after the earthquake. The pre-positioning of materials at 20 major airfields across the globe has been suggested as a way to expedite the arrival of relief [9]. Pre-positioning requires substantial maintenance costs and a high risk of waste as medical and food supplies expire, but the concept has value, particularly for surgical supplies. Several agencies could have a pre-agreement to maintain these and share their use if a disaster were to arise. Some equipment, such as oxygen and medicines (particularly opiates for pain relief), may be subject to importation delays; the pre-positioning of essential supplies could be linked to international customs pre-clearance that would allow rapid importation.

## Supply

In a mass disaster, airports become congested and the short supply of cargo planes often go to the highest bidder [9]. In Haiti, humanitarian cargo planes were diverted from Port-au-Prince to Santo Domingo, resulting in delays of essential surgical supplies such as an inflatable tent hospital brought in by MSF to assist with rapid surgical care, while high-profile media and visiting dignitaries were allowed to land at the US military–controlled Port-au Prince airport. Delays, losses, or incomplete reception of critical and sensitive surgery, anesthesia, and laboratory equipment hampered the overall effectiveness of the early response.

Essential humanitarian supplies should receive priority in any major crisis. Surgical care requires a certain amount of infrastructure that cannot be realistically maintained in every country such as sterilization machines, operating room equipment, surgical material, and instruments. “Portable operating room” kits that contain the most essential material needed to begin surgery can be hand-carried by the first wave of surgeons and anesthesiologists.

## Human Resources

Specialists such as surgeons, anesthesiologists, traumatologists, and emergency medicine doctors experienced in treating war wounded as well as working in resource-limited and disaster settings are rare [10]. Lists of these qualified specialists should be shared among relief agencies, in addition to other key staff such as scrub technicians, peri-operative nurses, wound nurses, physiotherapists, and psychologists. The American College of Surgeons had lists of hundreds of surgeons ready to be deployed to Haiti, but humanitarian agencies were reluctant to use them because inexperience in emergency settings can be more of a liability than a help. More recently, in the United Kingdom a register of surgeons, anesthesiologists, emergency physicians and nurses, and supporting staff qualified and willing to work in disasters has been established [11]. Training for disasters is provided by organizations such as the ICRC who lead 3-day war surgery seminars (which can be applied to natural disasters) and courses in disaster management. These are catered for surgeons with little to no disaster management experience, and also include lectures on humanitarian law and human rights that are beneficial to medical staff intending to work in disasters. ICRC also maintains several training hospitals worldwide [12]. It is imperative that surgical personnel interested in volunteering in humanitarian crises are effectively prepared or have prior field experience.

## Preparing for the Next Disaster

While efforts to coordinate the multitude of actors responding to the vast needs in emergencies will remain an ongoing concern of host governments and the UN, one of the most important lessons to be drawn from Haiti is that not all actors are equal, and organizational triage is needed to support prioritization of relief according to the most urgent needs. Given the particular challenges to delivering timely surgical assistance in emergencies, we propose the formation of the Emergency Surgery Coalition (ESC), a group consisting of organizations with extensive experience in delivering surgical care in man-made or natural disasters, and logistical and human resource capacity to deliver care worldwide at immediate notice. The ESC would facilitate coordination among major surgical providers to maximize the effectiveness of each actor's ability to deliver assistance in a given context. The ESC would not limit the reactivity and independence of its member organizations, and would be governed on a rotating basis by major humanitarian surgical providers who meet on a regular basis to ensure updated supplies and organizational and educational systems. Recommendations for the next disaster are summarized in Box 1.

### Prior to a Disaster

Prior to a disaster, the ESC could develop and maintain emergency prep kits worldwide. A continuous supply of medications such as analgesics, anesthetics, and antibiotics are needed and the ESC could work directly with the relevant government authorities and other competent bodies to ensure their expeditious delivery. It would prioritize the provision of training for surgical personnel, especially orthopedic and plastic surgeons, to prepare them for emergency work as an important step towards building a larger trained workforce. It could circulate lists of qualified surgical personnel among its members.

The ESC could also define model surgical strategies and triage plans for major disasters. When thousands of victims need surgical attention, not all can be assisted when resources are limited. In Haiti, most surgical agencies provided care without a specific strategy and resources may not have been utilized as efficiently as possible. For example, some surgeons spent precious hours trying to save the life of one patient at the expense of treating several others who were less gravely injured.

Finally, in order to improve program monitoring and evaluation, the ESC could encourage its members to agree upon a standard database with standard typology, as has been promoted by other organizations [13].

### During the Disaster

A needs assessment in the first hours after the disaster is critical to evaluate the level of response needed and should be conducted by agencies already present at the time of the disaster if possible. The ESC could rapidly disseminate this information to other major providers to facilitate planning.

In a disaster setting, the ESC would improve referral and transfer systems. While there is a need for many triage and stabilization points and a critical number of operating theatres, specialty services such as intensive care units should be limited in number and centralized. For example, in Haiti, one organization provided dialysis for crush syndrome and patients from all over the city were transferred for this service [14]. Without an overall coordinating body, many agencies try to provide all levels of care, resulting in duplication of some services and a paucity of others.

### Post Disaster

There is a pressing need for better monitoring and evaluation of surgical programs in humanitarian settings. The establishment of an inter-agency database would permit an analysis of trends and risk factors related to mortality and morbidity rates associated with surgical disease and interventions, and allow for interventions to be adapted accordingly in future disasters.

Efforts must also be made to better characterize the burden of surgical disease in crisis settings. Reliable estimates are lacking in general [15], and in humanitarian emergencies in particular [16]. While the nature and volume of medical need in humanitarian emergencies is difficult to predict, concerted efforts have led to the establishment of reliable datasets for the analysis of trends in mortality and morbidity due to infectious diseases, malnutrition, and violence 17. Recent initiatives to better estimate the global burden of surgical disease could help to better characterize the surgical burden of disease in humanitarian crises.

## Conclusions

Improved surgical delivery will require pre-emptive planning and inter-agency coordination. The ESC, comprising key players from major surgical humanitarian agencies, is a proposal to improve surgical delivery in emergencies and could support collaboration between surgical actors in an effort to minimize delay and duplication in the deployment of essential surgical services in future disasters.

## Abbreviations

ESC: Emergency Surgery Coalition
ICRC: International Committee for the Red Cross
MSF: Médecins sans Frontières
UN: United Nations
